# Radiographic and ultrasonographic findings in a dog with emphysematous pyometra

**DOI:** 10.1186/s13028-018-0419-z

**Published:** 2018-10-29

**Authors:** Chiara Mattei, Martina Fabbi, Kerstin Hansson

**Affiliations:** 1 University Animal Hospital, Swedish University of Agricultural Sciences, Ultunaallén 5A, Box 7040, 75007 Uppsala, Sweden; 2 Dipartimento di Scienze Medico Veterinarie, University of Parma, Parma, Italy; 3 Department of Clinical Sciences, Swedish University of Agricultural Sciences, Uppsala, Sweden

**Keywords:** Dog, Emphysematous, Gas, Pyometra, Radiography, Ultrasound, Uterus

## Abstract

**Background:**

Emphysematous pyometra is a rare canine disease characterized by gas-forming bacteria infecting the uterus and causing an accumulation of both gas and infectious exudate in the uterine lumen. While radiological features of emphysematous pyometra have been previously described in dogs, the ultrasonographic appearance has not been reported.

**Case presentation:**

A 7-year-old intact female Labrador Retriever was presented because of a 1 day history of vomiting, anorexia, mild polyuria/polydipsia and signs of fatigue. On physical examination the dog had a swollen vulva with a sparse amount of yellow discharge. Lateral and ventrodorsal radiographs showed a dilated predominantly gas-filled tubular structure located in the mid and cranial abdomen traversing from left to right and ending dorsally at the level of the 12th thoracic vertebra. A small intestinal ileus was initially suspected. Following the radiographic examination, abdominal ultrasound was performed. In the left mid and caudal abdomen there were two thin-walled gas-containing tubular structures. One had the typical layered appearance of an intestinal wall and represented the descending colon. The second structure had a similar thickness but homogenously hypoechoic wall and contained gas and echogenic fluid in the lumen. By use of several positional changes of the dog aiming to alter the location of the intraluminal gas, the second structure was traced to the right ovary cranially and the uterine body caudally, confirming that the structure was the right uterine horn. A final diagnosis of emphysematous pyometra was made.

**Conclusion:**

Ultrasound can be used as a non-invasive diagnostic method to differentiate between small intestinal ileus and emphysematous pyometra.

## Background

Emphysematous pyometra in the dog is a rare, life threatening disease, characterized by gas-forming bacteria infecting the uterus and causing an accumulation of both gas and infectious exudate in the uterine lumen [1]. To date, only six case reports have been described in dogs [1–6], and they all focus on the radiological features of emphysematous pyometra, which include large tubular structures containing gas or mixed gas and soft tissue/fluid opacities. The main differential diagnosis for emphysematous pyometra is small intestinal ileus. The previously reported radiological cases were conclusive mainly because of the bilateral involvement of the uterine horns, that created a bifurcating gas-filled tubular structure, or because of the severe degree of distension, that resembled a uterine horn rather than a small intestinal loop. Contrast radiography (upper gastrointestinal and barium enema) was also successfully used in some cases [4, 5] to distinguish between uterus and intestines.

We describe an unusual case of emphysematous pyometra, predominantly affecting one uterine horn that was in an unexpected anatomical position.

## Case presentation

A 7-year-old intact female Labrador Retriever was presented because of a 1 day history of vomiting, anorexia, mild polyuria/polydipsia and signs of fatigue. The owner had noticed some discharge from the vulva, as well as mucus and helminths in the feces. The dog had been in estrus 2 weeks before presentation but was not mated. The owner reported episodes of vomiting and weakness during the dog’s previous estrus cycles. On physical examination the dog was normothermic, had a swollen vulva with a sparse amount of yellow discharge and showed signs of pain on abdominal palpation. Hematology showed mild leukocytosis (18.96 × 10^9^ cells/L, reference 5.05–16.76 × 10^9^ cells/L). A serum chemistry panel identified mild metabolic hypochloremia and respiratory alkalosis and mildly elevated lactate.

### Diagnostic imaging

**Fig. 1:**
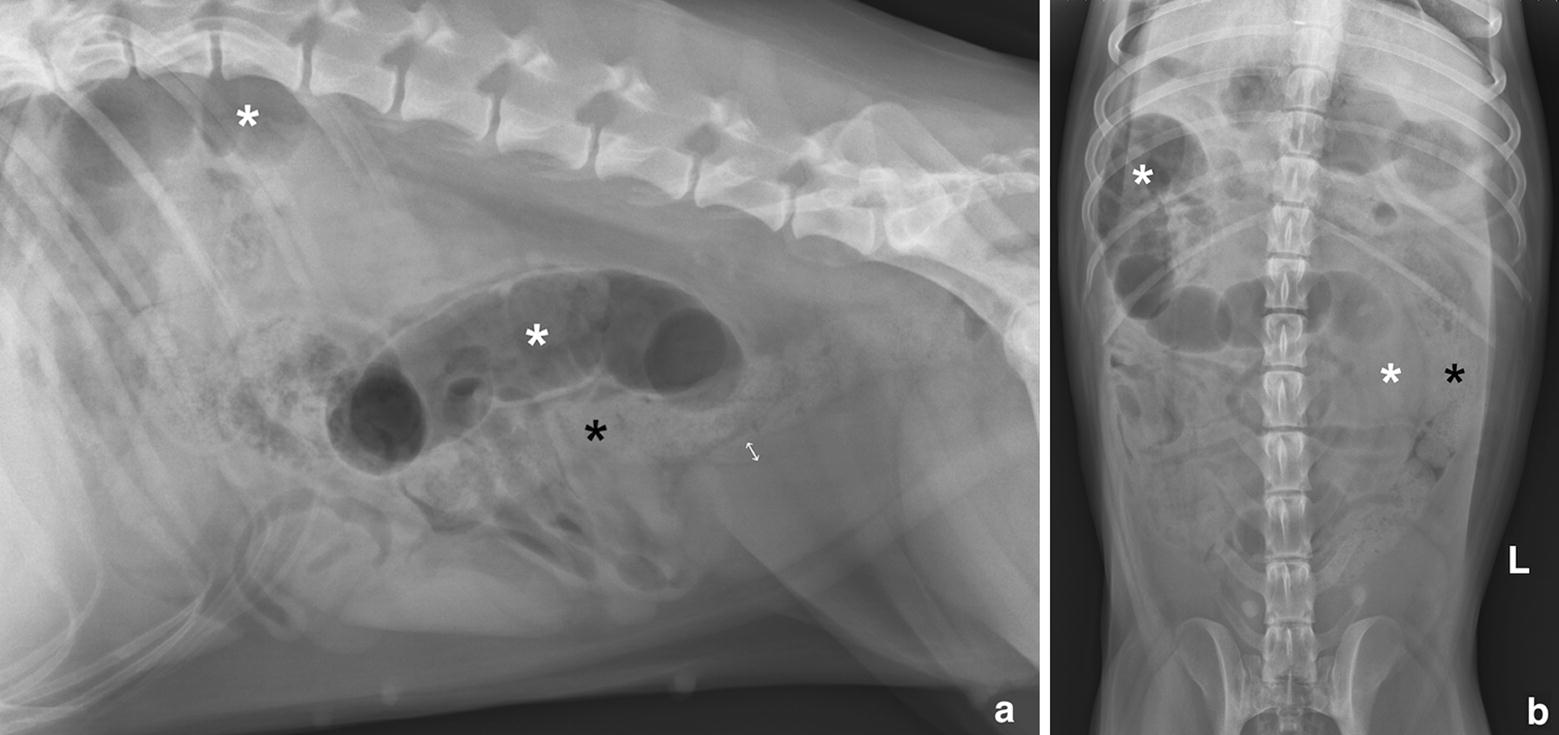
Left lateral (**a**) and ventrodorsal (**b**) abdominal radiographs. The mainly gas-filled and partly fluid-filled tubular structure (white asterisks) is seen dorsally (**a**) and medially (**b**) to the descending colon (black asterisk), extending to the right craniodorsal abdomen. The uterine body is faintly seen summating over the craniodorsal aspect of the urinary bladder (**a** double-headed arrow) but the uterine horns cannot be followed. The letter L in b indicates the left side of the dog

Left lateral (Fig. 1a) and ventrodorsal (Fig. 1b) abdominal radiographs were obtained. The lateral radiograph showed two gas-filled tubular structures, measuring up to 3.5 times the height of the body of the 5th lumbar vertebra. There was one gas-filled tubular structure in the central abdomen, dorsal and parallel to the descending colon, and one in the craniodorsal abdomen, just ventral to the caudal thoracic and cranial lumbar vertebrae. The ventrodorsal radiograph showed that the two gas-filled structures were parts of the same, slightly contracted, tubular structure. In the caudal and mid abdomen the tubular structure was medial to the descending colon and had a soft tissue/fluid opacity in this region. The tubular structure then turned to the right crossing the midline at the level of the two first lumbar vertebrae. The most cranial segment followed the right cranial abdominal/caudal thoracic wall to reach the most dorsal part of the right cranial abdomen. The difference in location of the intraluminal gas on the lateral and ventrodorsal radiograph was considered to be due to gravity as a result of positional changes of the dog. Thus, the tubular gas and fluid-filled structure could be followed almost the entire length of the abdomen, from the cranial aspect of the urinary bladder to the stomach. In the caudal abdomen on the lateral radiograph the uterine body was faintly visible between the descending colon and the urinary bladder, measuring approximately 1.3 cm in diameter, subjectively considered to be normal for the large size of the dog and the phase in the estrus cycle. Small intestines with normal diameter and content were seen in the mid-abdomen.

Because of the position and the gas content in the structure, the main radiological suspicion was small intestinal ileus likely due to mechanical intra- or extraluminal obstruction, despite that no foreign body or mass could be seen.

**Fig. 2:**
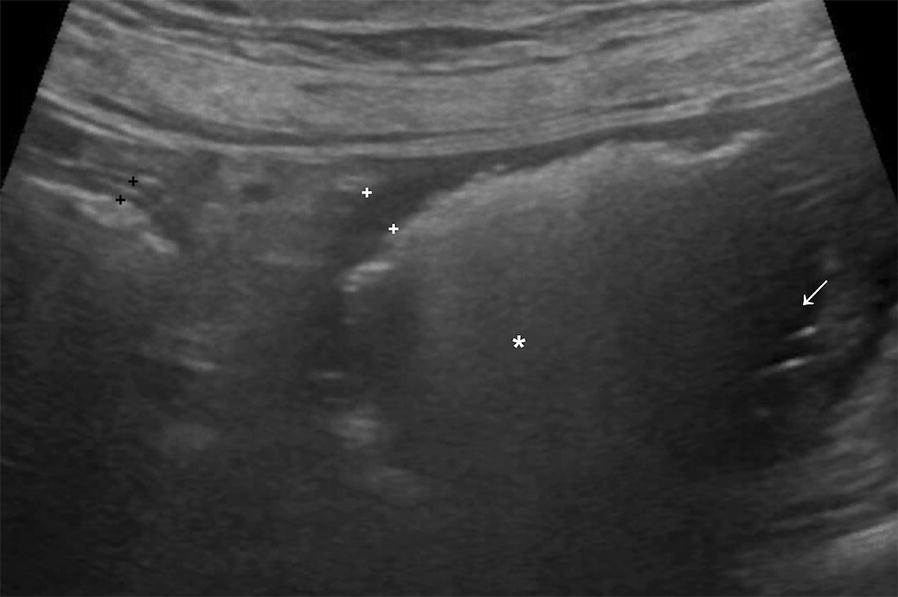
Ultrasound scan of the caudal abdomen, transverse plane (left of the image is the right of the dog). This close-up image allows the differentiation between the walls of the two tubular structures. On the left side a layered wall of the colon (black cursors) and on the right side a homogenously hypoechoic wall of the uterus (white cursors). Note that some fluid content can be seen in the uterus (arrow) which is otherwise gas-filled (asterisk)

**Fig. 3:**
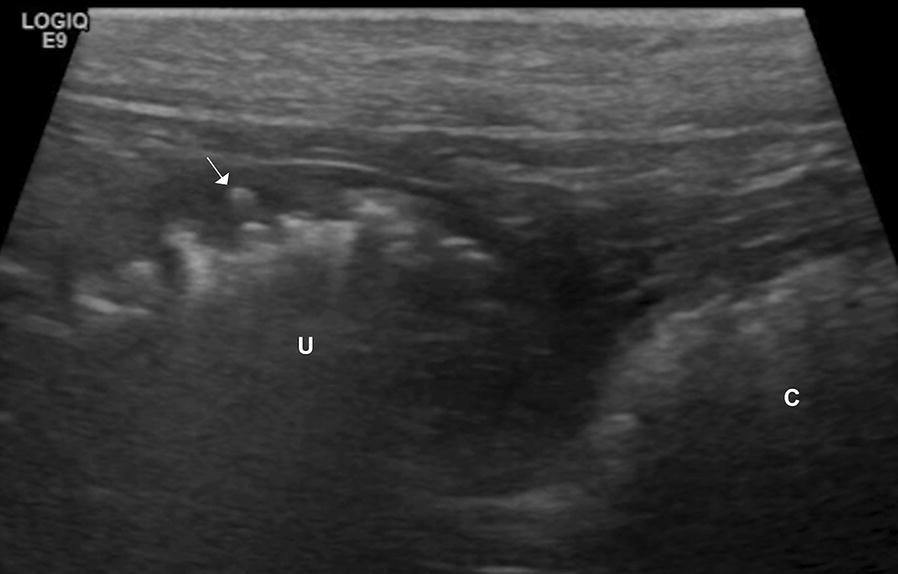
Ultrasound image in a sagittal plane of the caudal abdomen (left of the image is cranial). The uterus (U) is on the left of the image and the colon (C) is on the right. Note the uneven uterine luminal surface, with hyperechoic speckles in the wall (arrow), likely indicating infiltration of gas in the mucosa, indicative of emphysema or ulceration

Following the radiographic examination, abdominal ultrasound was performed to confirm ileus and locate the suspected obstruction. In the left mid and caudal abdomen there were two thin-walled tubular structures whose content created a hyperechoic interface associated with reverberation and comet tail-artifacts, indicating gas content (Figs. 2 and 3). One of these structures had the typical appearance of an intestinal wall, with alternating hypo- and hyperechoic layers, and in some parts the interface with the content created a dirty acoustic shadow. This structure was considered to represent the descending colon. A second structure had a similar thickness but homogenously hypoechoic wall, without visible layers. The interface between the wall and the luminal content was uneven and, in some parts, hyperechoic speckles were visible within the wall, creating a faint “comet-tail” artifact, suspected to be gas within the wall, consistent with emphysema of the wall or ulceration (Fig. 3). Apart from the gas there was echogenic fluid in the lumen in the second structure, visible when the gas was moving. When tracing the second structure, it followed the path of the colon but was medial to the descending and ascending colon and caudal to the transverse colon. By use of several positional changes of the dog aiming to change the location of the intraluminal gas and any superimposition of other organs, the structure could be seen reaching the right ovary from the cranial aspect, while caudally it was connected to the uterine body, confirming that this was the right uterine horn. The maximum diameter of this right uterine horn was 3.3 cm. In order to make it possible to follow the left uterine horn, positional changes of the dog were required to move the right horn from its location in the left hemiabdomen. The left uterine horn was 0.9 cm in diameter, with mild amounts of intraluminal fluid and gas. The right medial iliac lymph node was mildly hypoechoic and rounded compared to the left one, with a thickness of 2 cm, interpreted as reactive lymphadenopathy. No free fluid nor free gas were found in the abdomen. The rest of the abdominal organs were normal.

The radiological diagnosis was emphysematous pyometra, predominantly affecting the right uterine horn.

### Outcome

**Fig. 4:**
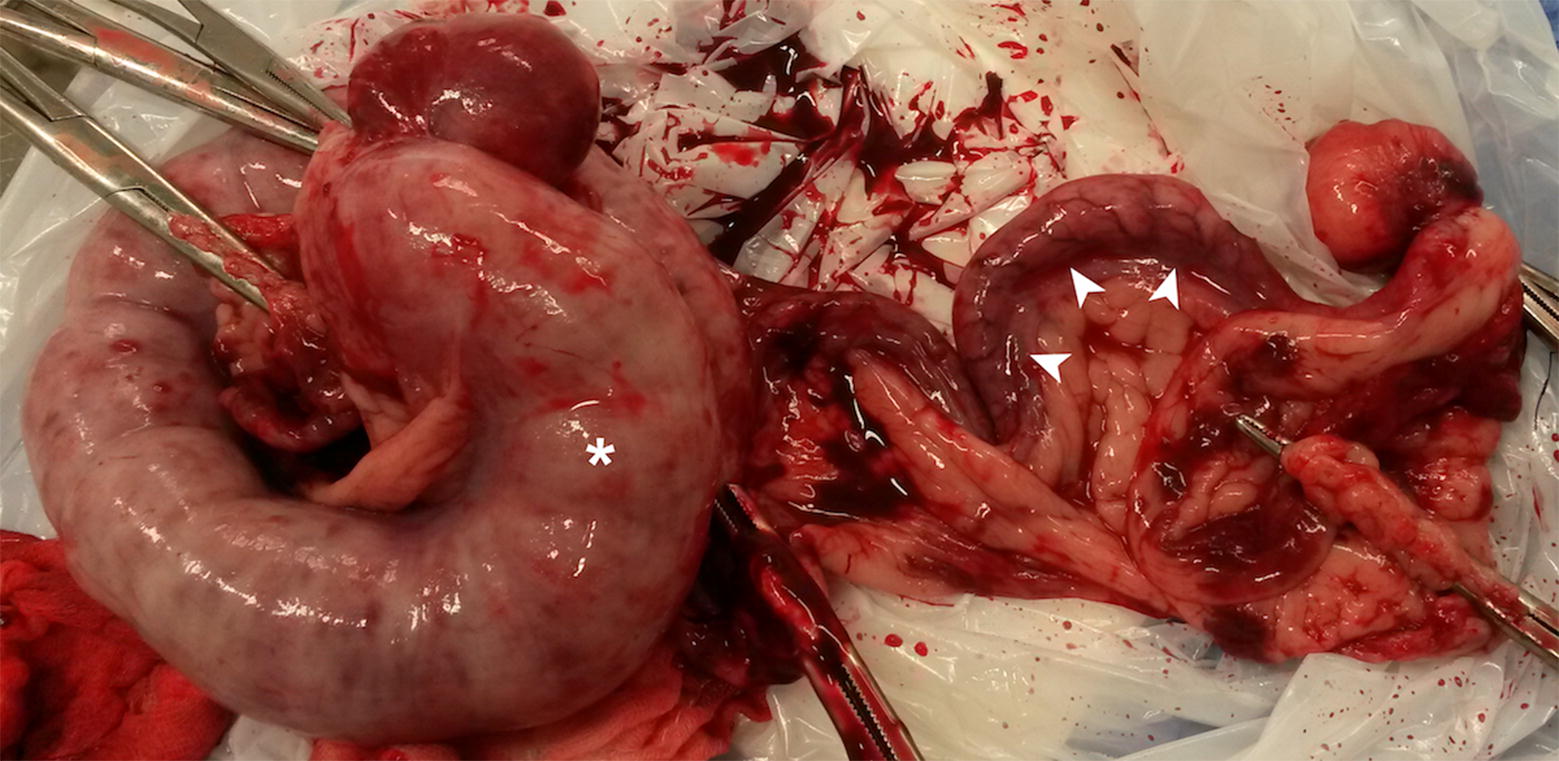
Post-surgical gross appearance of the uterus. The right horn is on the left of the image (asterisk). Arrowheads are pointing to parts of the left uterine horn

The dog underwent surgery for ovariohysterectomy immediately after being treated with supporting intravenous Ringer-acetate solution (Fresenius AG, Bad Homburg, Germany) and methadone (Meda AB, Solna, Sweden). The ultrasonographic findings were confirmed on surgery (Fig. 4). The right horn measured up to 5 cm in diameter and was thin-walled, distended and fluctuant due to the gaseous and liquid content. The left horn measured 1 cm in diameter and contained mainly fluid. When cutting through the uterine wall into the lumen gas and purulent exudate were found. Fluid samples for aerobic and anaerobic bacterial cultures were taken and *Escherichia coli* and beta-hemolytic streptococci were isolated. The uterus was not submitted for histopathology. The other abdominal organs were grossly unremarkable. The patient was treated with antibiotics in accordance to the result of the antibiogram and recovered fully in 2 weeks.

## Discussion and conclusions

Pyometra is a bacterial infection that leads to accumulation of pus in the uterus, and commonly affects intact female dogs. Its pathogenesis is related to the repeated exposure of the endometrium to progesterone during the long luteal phase of the estrus in bitches [7]. The progesterone-sensitized uterus is suitable not only for pregnancy, but also for bacterial infection, because progesterone stimulates endometrial glandular secretions, induces functional cervical closure, decreases myometral contraction and suppresses local immune responses [7]. *Escherichia coli* is the most commonly isolated organism [7] but a wide range or other species have also been found [1, 2, 7–9]. Rarely the uterus is colonized by gas-producing bacteria, which lead to emphysematous pyometra. In previous cases of canine emphysematous pyometra *Staphylococcus* spp., *Pseudomonas aeruginosa*, *Citrobacter diversus*, *Clostridium perfringens* and *Enterococcus avium* have been isolated [1–5].

The radiological features of emphysematous pyometra have been described in dogs [1–5], and include tubular structures containing gas or mixed gas and soft tissue/fluid, which had to be differentiated from small intestinal gas [10]. In the previously published case reports, the radiological studies were conclusive mainly because of the bilateral involvement of the uterine horns, creating a bifurcating gas-filled tubular structure, typical for the uterus, or for the severe degree of distension, that fit more a uterine horn than a small intestinal loop. Upper gastrointestinal and barium enema contrast radiography was successfully used in some cases [4, 5] to distinguish between uterus and intestines.

In cases of pyometra, the uterine horns are usually symmetrically affected, but this is variable and only one horn may be affected [10]. The described case was misleading because only one horn was significantly enlarged and fluid and gas-filled, while the other horn was only mildly affected. The primary radiological diagnosis was a segmental dilated small intestine, since the gas-filled structure could not be clearly connected to the uterine body and the position was atypical for a distended uterine horn. The unusual location of the right uterine horn was probably because of the gas content causing it to be more fluctuant and moveable compared to the classic fluid-filled heavy horns. Furthermore, the contracted appearance of the tubular structure was interpreted as an increased intestinal peristalsis, possibly related to obstruction, another feature more typical for the gastrointestinal than the genital tract.

The ultrasonographic appearance of a pathologically altered uterus varies, but luminal fluid is commonly found and ultrasound is of great use to detect mild accumulation of fluid, even if differentiation of the type of fluid is not possible [11].

In this case the ultrasound examination was performed to localize the area of and the reason for the suspected obstruction (foreign body, mass, focal functional ileus) since this could not be established based on the radiographs. Usually ultrasound gives limited information when a large amount of gas is present in the investigated organ, since gas is a strong reflector and prevents transmission of ultrasound waves, creates reverberation artifacts and inhibits diagnostic information from being obtained [12]. Despite the gas content, the wall of the hollow structure could be clearly evaluated and gave valuable information. The wall was not typical for an intestinal loop, since the layered pattern was missing. The proximity of the structure to the colon highlighted the different wall appearance, homogenously hypoechoic for the uterine wall, histologically composed of a mucosa and a muscularis, which cannot be differentiated with ultrasound [13], and alternating hyper- and hypoechoic layered for the intestinal wall, which is normally differentiated with ultrasound.

Severe inflammatory bowel disease can significantly affect the intestinal wall layers, even causing their complete loss, thereby making it difficult to differentiate between an abnormal intestinal segment and a gas-filled uterus. The peristaltic movement can help distinguish intestinal tract from genital tract, but altered motility is often present with severe enteritis, making this parameter less useful for distinguishing between intestines and a gas-filled uterus. Crucial to this case was the continual change in position of the dog during the examination, that allowed both the gas in the uterus to move and the superimposed colon to change position. These positional changes of the dog were repeated until a clear connection of the tubular structure with the right ovary and uterine body was identified, allowing for the definitive diagnosis.

In conclusion, as previously stated [5], radiological differential diagnosis of a dilated gas-filled structure in an intact female dog has to include emphysematous pyometra, even when no clear connection to the uterine body can be seen and the unusual position and unilateral affection of the organ seem to make this diagnosis less likely. In cases of uncertain radiological diagnosis, ultrasound represents a non-invasive and relatively rapid diagnostic method for differentiating between small intestinal ileus and emphysematous pyometra. A gas-filled hollow organ with thin, uniformly hypoechoic wall, lacking the typical intestinal layers and peristaltic movements, should raise the suspicion of emphysematous pyometra. To confirm the diagnosis of emphysematous pyometra the gas-filled structure should be clearly connected to the uterine body or ovaries.
